# Supplementary figures and images for: NLRP7 plays a functional role in regulating BMP4 signaling during differentiation of patient-derived trophoblasts
Source: Cell Death Dis. 2020 Aug 19;11(8):658. doi: 10.1038/s41419-020-02884-1 (PMC7438493; doi:10.1038/s41419-020-02884-1)

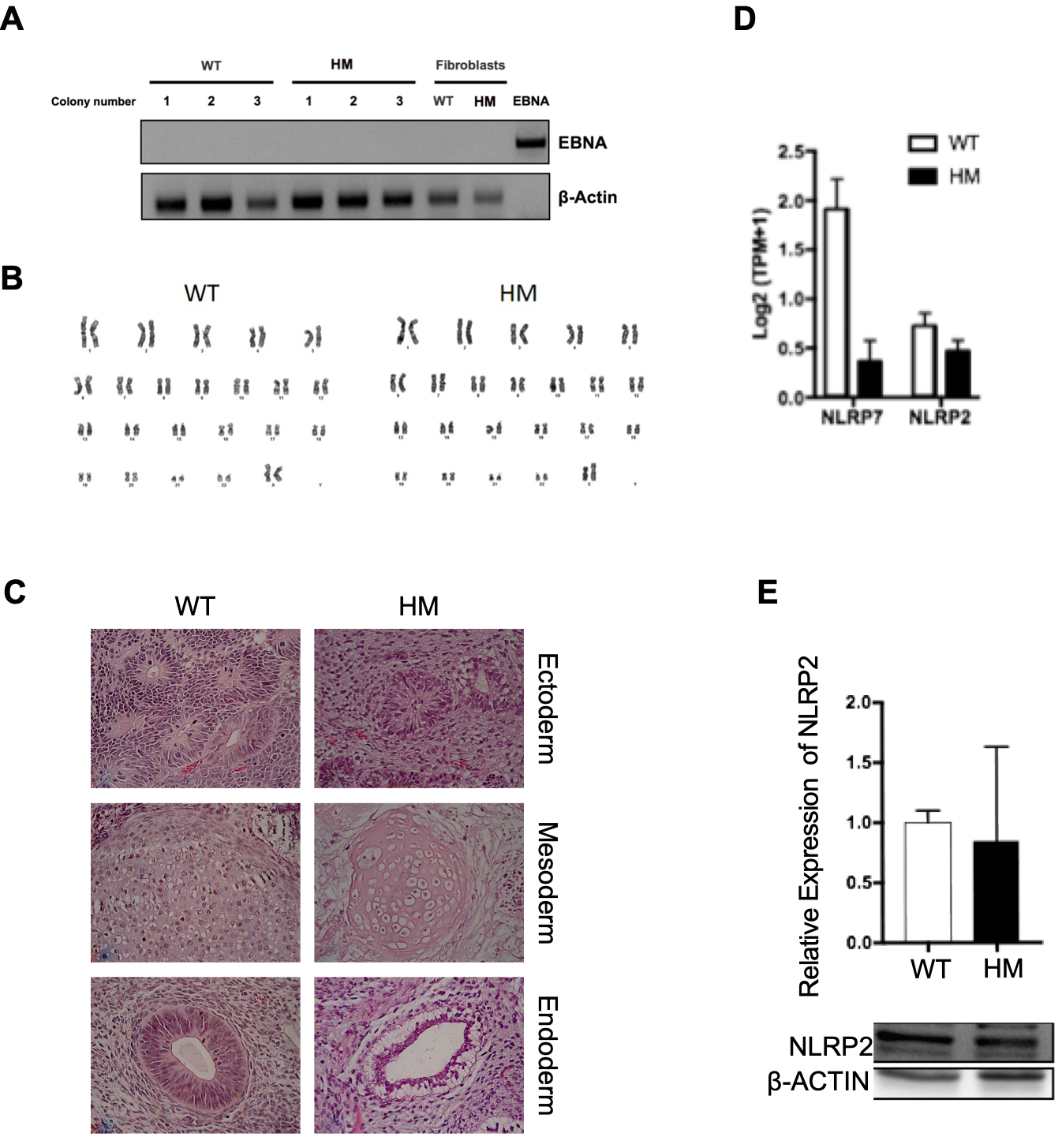

Supplement: Supplementary file 2 — Figure S1 [file 41419_2020_2884_MOESM2_ESM.png]

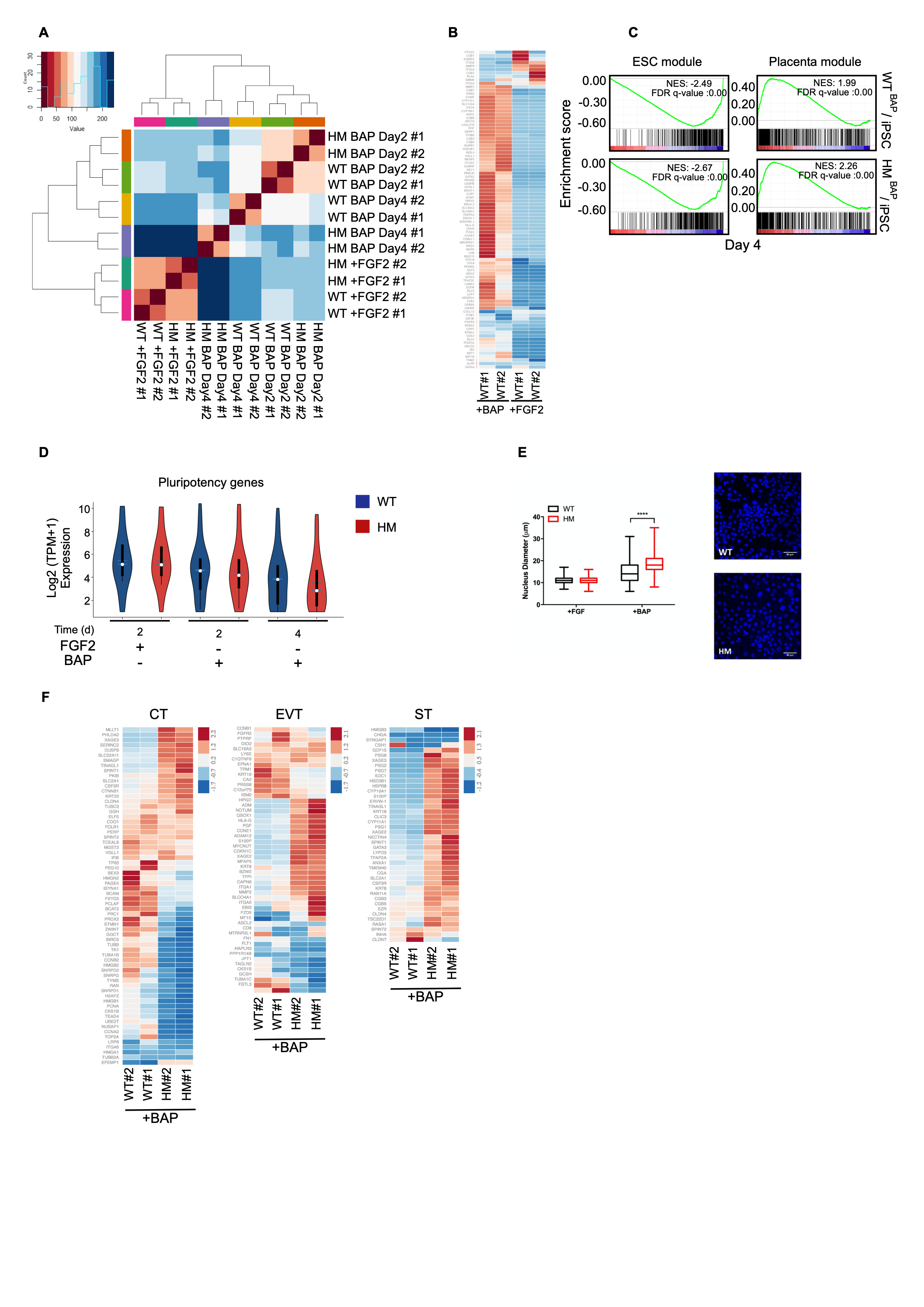

Supplement: Supplementary file 3 — Figure S2 [file 41419_2020_2884_MOESM3_ESM.png]

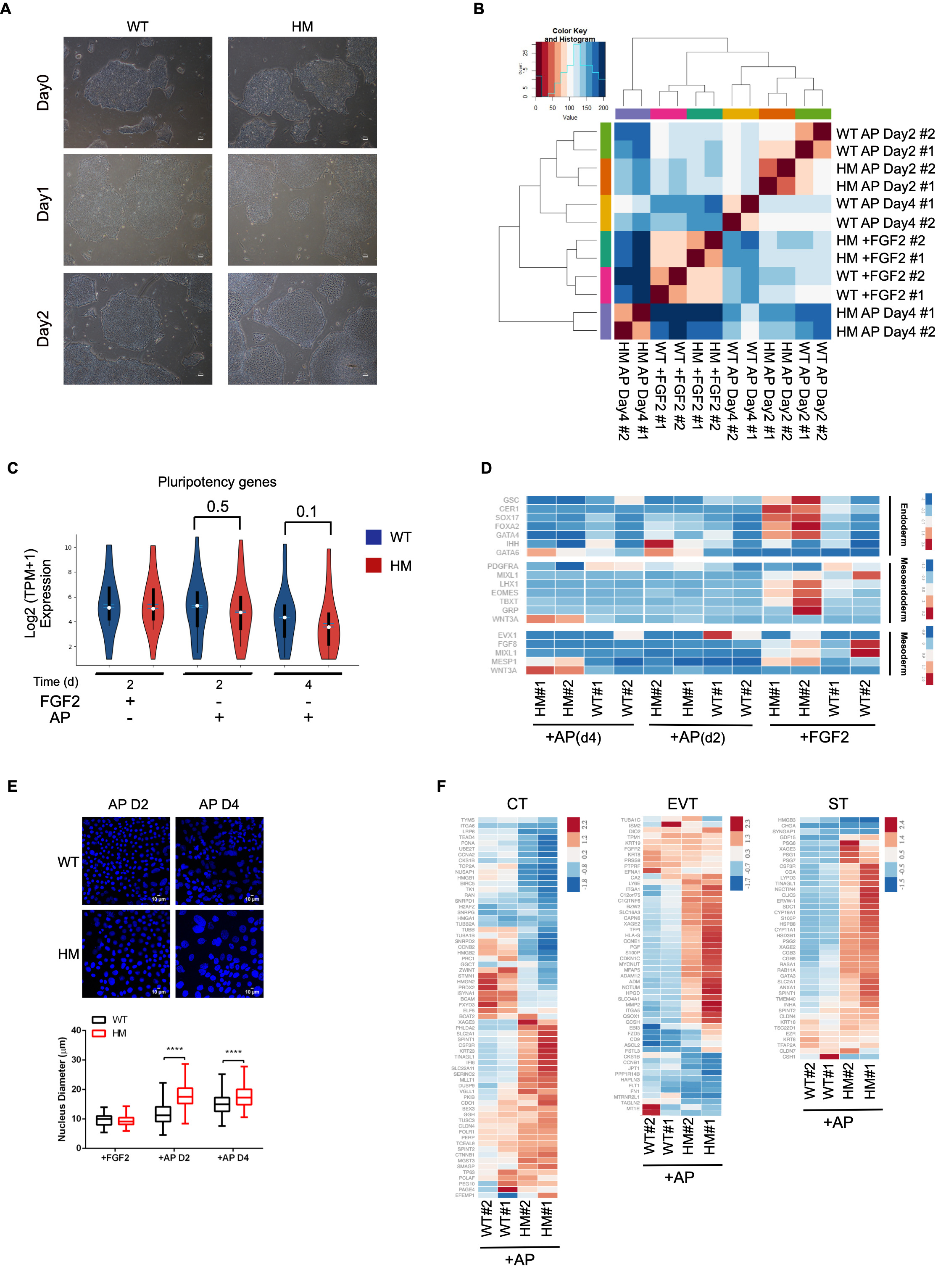

Supplement: Supplementary file 4 — Figure S3 [file 41419_2020_2884_MOESM4_ESM.png]

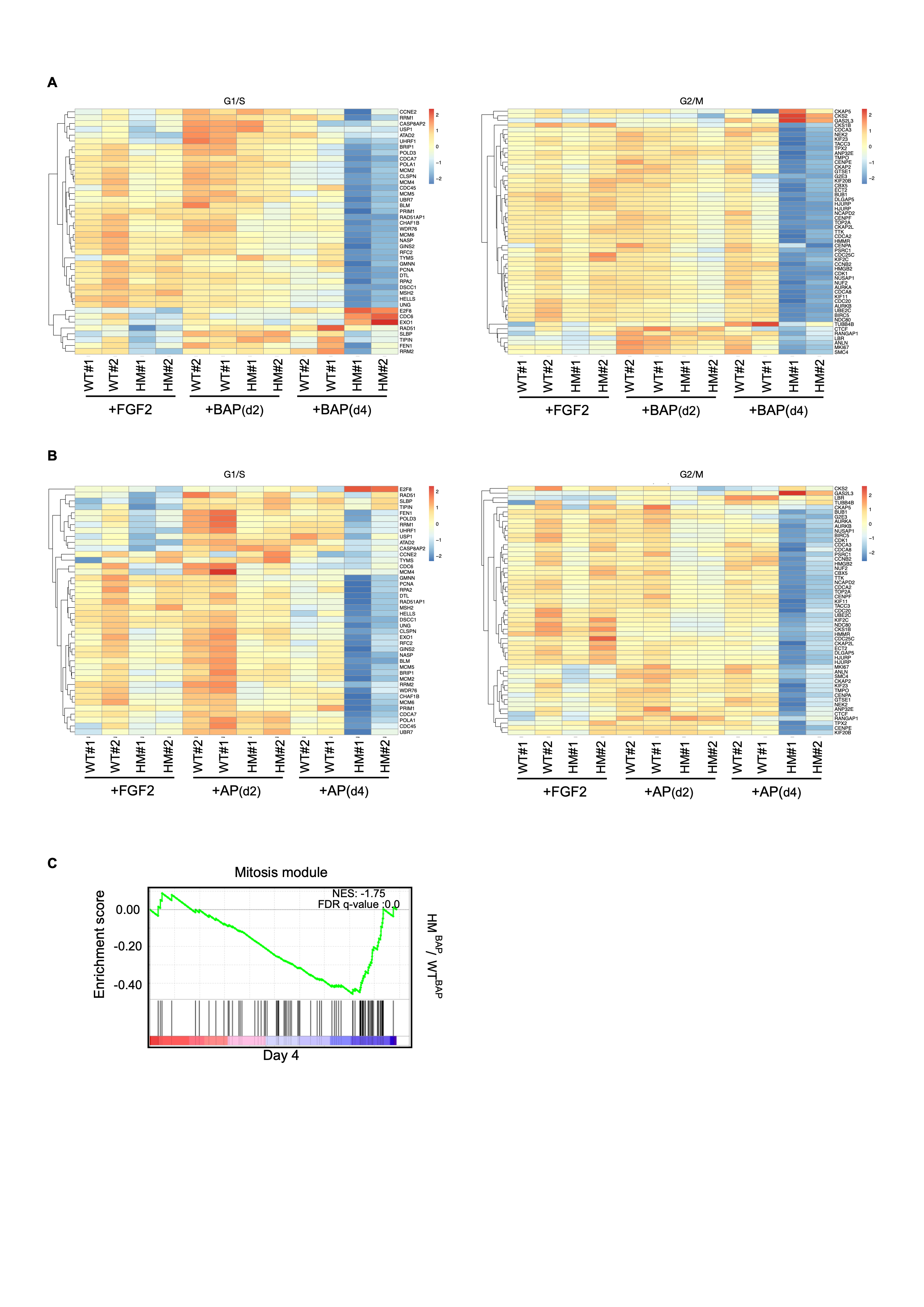

Supplement: Supplementary file 5 — Figure S4 [file 41419_2020_2884_MOESM5_ESM.png]

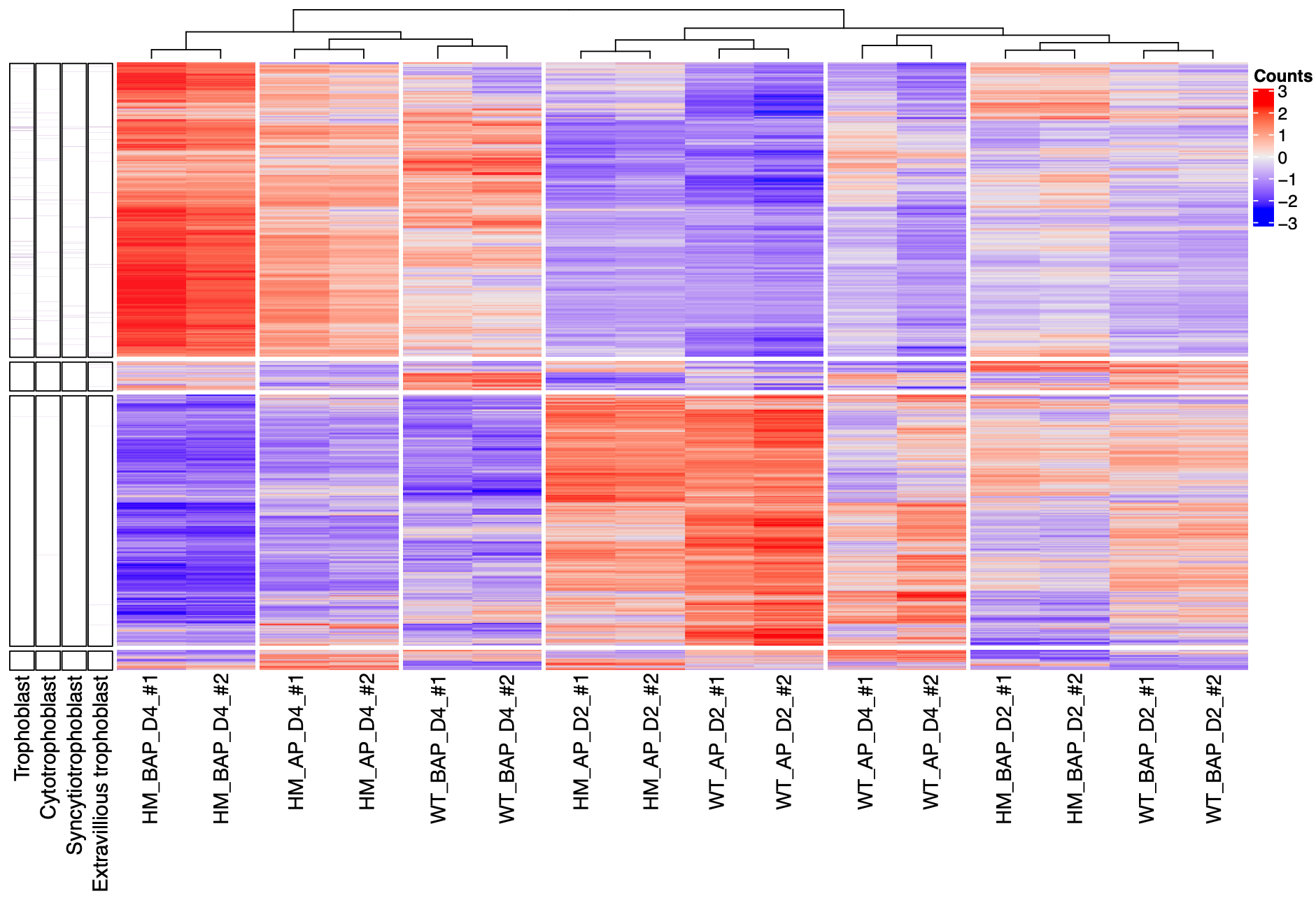

Supplement: Supplementary file 6 — Figure S5 [file 41419_2020_2884_MOESM6_ESM.png]

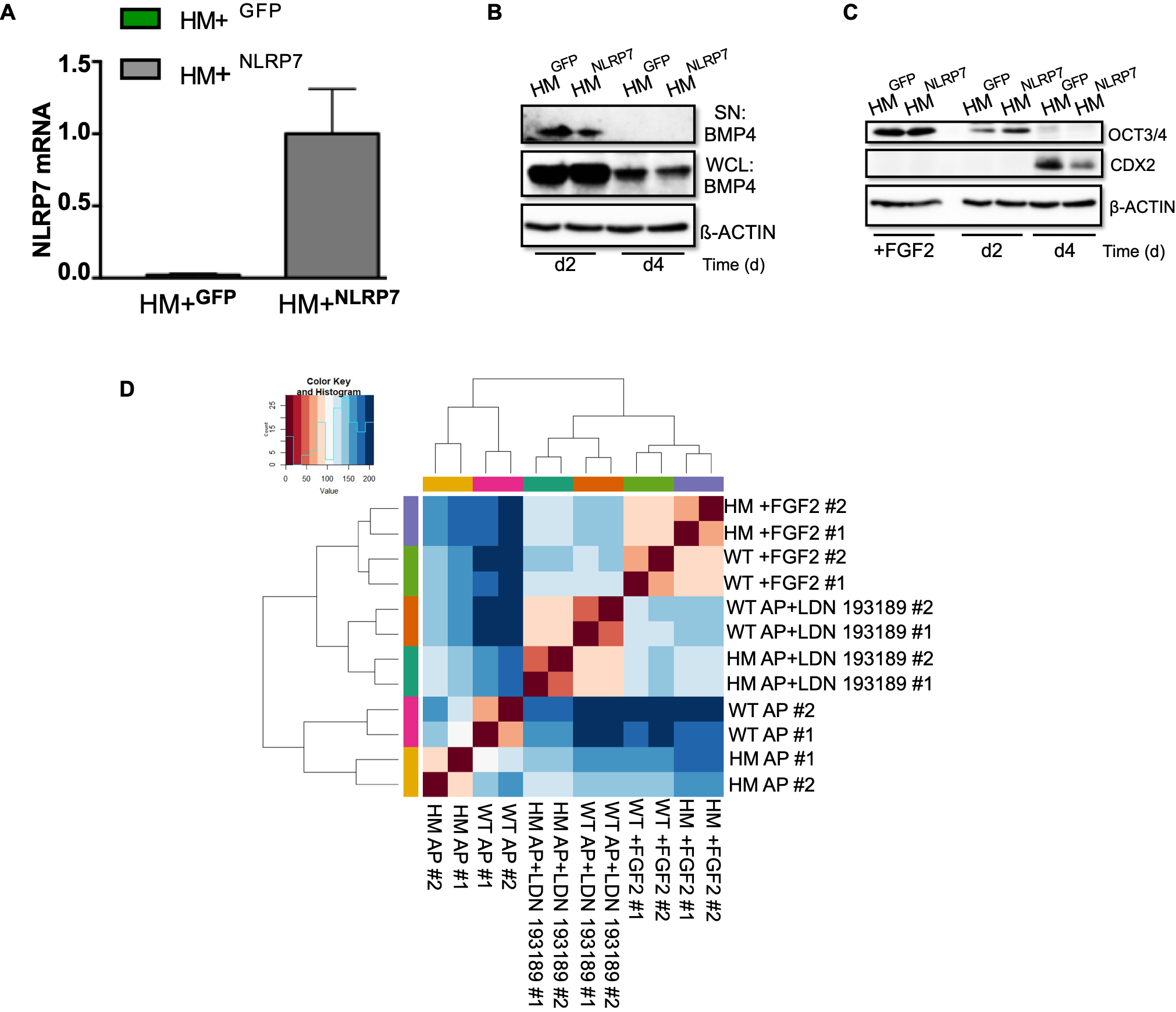

Supplement: Supplementary file 8 — Figure S7 [file 41419_2020_2884_MOESM8_ESM.png]
