# Supplementary material for: NLRP7 plays a functional role in regulating BMP4 signaling during differentiation of patient-derived trophoblasts
Source: Cell Death Dis. 2020 Aug 19;11(8):658. doi: 10.1038/s41419-020-02884-1 (PMC7438493; doi:10.1038/s41419-020-02884-1)
Supplement: Supplementary file 12 — Table 4 [file 41419_2020_2884_MOESM12_ESM.pdf]

qRT-PCR PRIMERS	Forward	Reverse
BMP4	5’TCCTGGTAACCGAATGCTGA	5’CCTGAATCTCGGCGACTTTT
CDX2	5′GCCAAGTGAAAACCAGGACG	5′TCCTCCGGATGGTGATGTAG
CGB	5’GTCAACACCACCATGTGTGC	5’GGTAGTTGCACACCACCTGA
GAPDH	5’GGAGCGAGATCCCTCCAAAAT	5’GGCTGTTGTCATACTTCTCATGG
HLA-G	5′CTCTCAGGCTGCAATGTGAA	5′CATGAGGAAGAGGGTCATGG
INSL-4	5’CCCCATGCCTGAGAAGACAT	5’GTTGTTGGAGGTTGACACCATT
NANOG	5’ CATGAGTGTGGATCCAGCTTG	5’CCTGAATAAGCAGATCCATGG
NLRP2	5’GCTGCTGTGTTGGTTGTCAG	5’GCAGTTCCAAAGCACCAAGG
NLRP7	5’TAAGGAATGCGACTGTGAACATC	5’TGCTAACTCCGAGTCTTCTTCT
PGF	5′TCCTACGTGGAGCTGACGTT	5′CACCTTTCCGGCTTCATCTTC
POU5F1	5′GGCTCGAGAAGGATGTGGT	5′GCCTCAAAATCCTCTCGTTG
PSG4	5’CCAGGGTAAAGCGACCCATT	5’AAGAATATTGTGCCCGTGGGTT
